# Subgrouping patients with zoster-associated pain according to sensory symptom profiles: A cluster analysis

**DOI:** 10.3389/fneur.2023.1137453

**Published:** 2023-02-17

**Authors:** Hee Jung Kim, Kyung Bong Yoon, Misun Kang, Yun Seok Yang, Shin Hyung Kim

**Affiliations:** Department of Anesthesiology and Pain Medicine, Anesthesia and Pain Research Institute, Yonsei University College of Medicine, Seoul, Republic of Korea

**Keywords:** zoster-associated pain, herpes zoster, cluster analysis, sensory symptoms, painDETECT

## Abstract

**Background and goal of study:**

Patients with zoster-associated pain exhibit a variety of sensory symptoms and forms of pain and complain of different pain patterns. The purpose of this study is to subgroup patients with zoster-associated pain who visited a hospital using painDETECT sensory symptom scores, analyze their respective characteristics and pain-related data, and compare similarities and differences among the groups.

**Materials and methods:**

The characteristics of 1,050 patients complaining of zoster-associated pain and pain-related data were reviewed retrospectively. To identify subgroups of patients with zoster-associated pain according to sensory symptom profiles, a hierarchical cluster analysis was performed based on the responses to a painDETECT questionnaire. Demographics and pain-related data were compared among all subgroups.

**Results and discussion:**

Patients with zoster-associated pain were classified into 5 subgroups according to the distribution of sensory profiles, with each subgroup exhibiting distinct differences in the expression of sensory symptoms. Patients in cluster 1 complained of burning sensations, allodynia, and thermal sensitivity, but felt numbness less strongly. Cluster 2 and 3 patients complained of burning sensations and electric shock-like pain, respectively. Cluster 4 patients complained of most sensory symptoms at similar intensities and reported relatively strong prickling pain. Cluster 5 patients suffered from both burning and shock-like pains. Patient ages and the prevalence of cardiovascular disease were significantly lower in cluster 1. Patients in clusters 1 and 4 reported longer pain duration compared with those in clusters 2 and 3. However, no significant differences were found with respect to sex, body mass index, diabetes mellitus, mental health problems, and sleep disturbance. Pain scores, distribution of dermatomes and gabapentinoid use were also similar among the groups.

**Conclusions:**

Five different subgroups of patients with zoster-associated pain were identified on the basis of sensory symptoms. A subgroup of younger patients with longer pain duration showed specific and distinct symptoms, such as burning sensations and allodynia. Unlike patients with acute or subacute pain, patients with chronic pain were associated with diverse sensory symptom profiles.

## Introduction

Cases of herpes zoster occur in at least 1 million people in the United States every year, and the incidence rate has been increasing worldwide over the past 20 years (1). Approximately one-fifth of herpes zoster patients report some pain at 3 months after the onset of the disease, and the incidence rate of postherpetic neuralgia (PHN) increases dramatically as patients age (2, 3).

Patients with zoster-associated pain, including herpes zoster infection and PHN, complain of diverse patterns of sensory symptoms despite the common cause of the pain. Some patients complain strongly of spontaneous pain, tingling sensations, and electric-shock-like pain, while others suffer from hypersensitivity to light touch or temperature. A simple and validated patient-reported screening questionnaire, painDETECT, can detect these characteristic sensory abnormalities (4, 5). Researchers have attributed this difference in pain patterns to the relative contributions of the peripheral and central mechanisms (6). However, no specific biomarkers have been found with mechanisms of zoster-associated pain. Therefore, subgrouping of patients according to sensory phenotype can be clinically important in patients with zoster-associated pain. Treatments that focus on similar phenotypes would improve individualized pain therapy and future clinical trial design (7, 8). Many investigators have expressed similar thoughts on this subject, and multiple studies have been conducted to identify the differences in the dynamics of the somatosensory system for some peripheral neuropathic pain conditions, including diabetic neuropathy and PHN (9–13). However, few studies have focused on zoster-associated pain from acute, subacute, and chronic pain conditions. We assumed that patients with zoster-associated pain who visit a hospital can be subgrouped into sensory profiles according to the somatosensory mechanism like other neuropathic pain.

The goal of this study is to classify patients with zoster-associated pain into groups that clearly represent each characteristic using a cluster analysis technique and to identify any similarities and differences in the characteristics of each cluster groups.

## Materials and methods

### Study population

This study was approved by the Institutional Review Board of Yonsei University Health System, Seoul, Republic of Korea (No. 4-2022-0819). As it is a retrospective observational study, the requirement for obtaining informed consent from the patients was waived. This manuscript complies with the STROBE checklists applicable to observational studies. Adult patients who complained of zoster-associated pain at their first visit from January 2016 to August 2021 were enrolled. Patients with incomplete medical records or those who did not complete the painDETECT questionnaire were excluded.

### Sensory symptom profiling using the painDETECT questionnaire

Sensory symptom profiles due to herpes zoster were described using the painDETECT questionnaire during the patient's first visit to our clinic. Patients complaining of neuropathic pain caused by herpes zoster freely described their symptoms and filled out the validated Korean version of the painDETECT questionnaire under the supervision of a researcher (14). The questionnaire consisted of 9 questions asking for sensory symptoms, pain course patterns, and radiating pain. Seven of the questions asked respondents to use a 0-to-5 Likert scale to describe pain quality associated with various sensory symptoms: burning sensations, tingling or prickling sensations, pain by light touch, electric shock-like pain, pain on cold/heat sensation, numbness, and pain by slight pressure (0 = never; 1 = hardly noticed; 2 = slightly; 3 = moderately; 4 = strongly; 5 = very strongly). To eliminate differences in individual pain-perception thresholds, an alternative value was obtained by subtracting the average value of all scores for each of the 7 questions. In this alternative score, values above 0 represent stronger sensations than individual average pain perception, and values below 0 represent less-intense sensations compared with individual average pain perception (10).

### Patient demographics and pain-related data measures

Patient characteristics and pain-related data were gathered from electronic medical records. Patient characteristics included age, sex, body mass index (BMI), presence of diagnosed comorbidities, and sleep disturbances. Diagnosed comorbidities included cardiovascular disease, diabetes mellitus, and mental health problems that required continuous medical intervention with regular hospital visits. Mental health problems were defined as those requiring psychiatric treatment or medication due to depression, anxiety, and stress over the past year. Average and maximum pain scores using a numeric rating scale (NRS), duration of pain, dermatomes, and the use of a gabapentinoid at the time of visit were identified as pain-related factors. The duration of pain was classified into < 1 month (acute), 1–3 months (subacute), and ≥ 3 months (chronic).

### Statistical analysis

#### Descriptive statistical analysis

Continuous variables were shown as mean ± standard deviation (SD), and categorical variables was tabulated using numbers (percentage). The normality of distribution was evaluated using a Shapiro–Wilk test. Differences among the cluster groups were analyzed using one-way analysis of variance and chi-squared tests. A Tukey's *post hoc* test or a chi-squared *post hoc* difference was used to determine the intergroup difference between the mean of the groups when significant differences existed. Statistical Package for the Social Sciences, version 26.0 (IBM Corp, Armonk, NY, USA) was used to analyze the data. A *P* < 0.05 was considered statistically significant.

#### Cluster analysis

**Figure 1:**
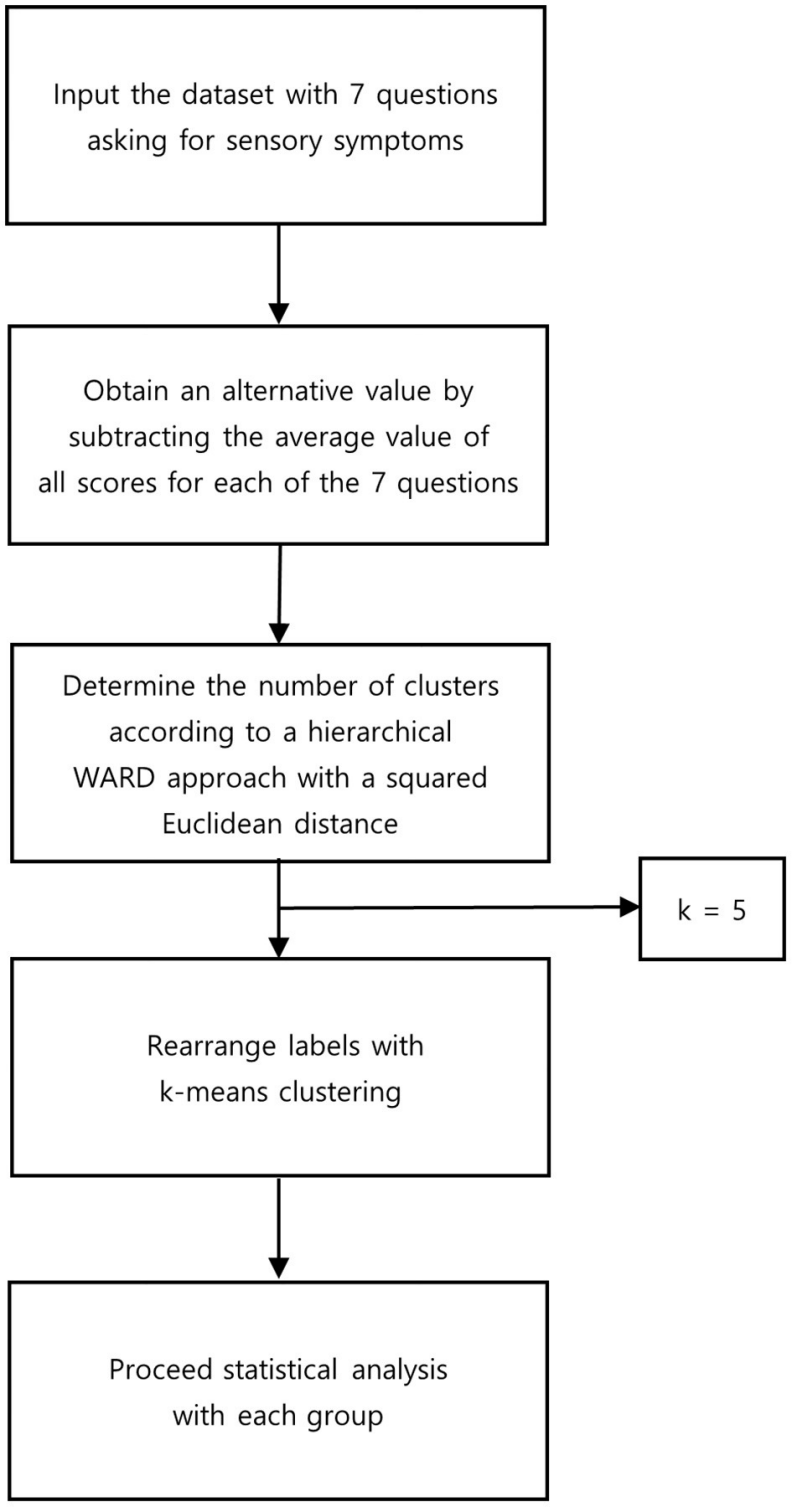
Flowchart for statistical methods.

A hierarchical cluster analysis was performed for subgrouping according to relevant sensory symptoms of zoster-associated pain (Figure 1). A hierarchical WARD approach with a squared Euclidean distance measure described previously (9, 10) was applied. Cluster analysis was conducted using the Python program, version 3.8.15 with Scikit-learn version 1.0.2. The cut-off point for the essential clusters was set at ~ 10% of the total cases. We chose to refer to a cut-off point of 5 clusters because solutions with fewer clusters could eliminate significant differences by the agglomerative clustering. A solution with five essential clusters was obtained by hierarchical WARD clustering as an optimal agreement on our decision criteria. To demonstrate the evidence for the solution and to rearrange the cases based on these results, a k-means cluster analysis was performed. This analysis led to equal results, which provides support for our chosen solution.

The cluster is represented by a pattern of questionnaire scores, indicating the typical pathological structure of each group. All profiles were given individually adjusted scores (see above). Heuristic interpretations of clusters were provided by experts. Statistical analysis was not performed because this was a heuristic approach.

## Results

**Figure 2:**
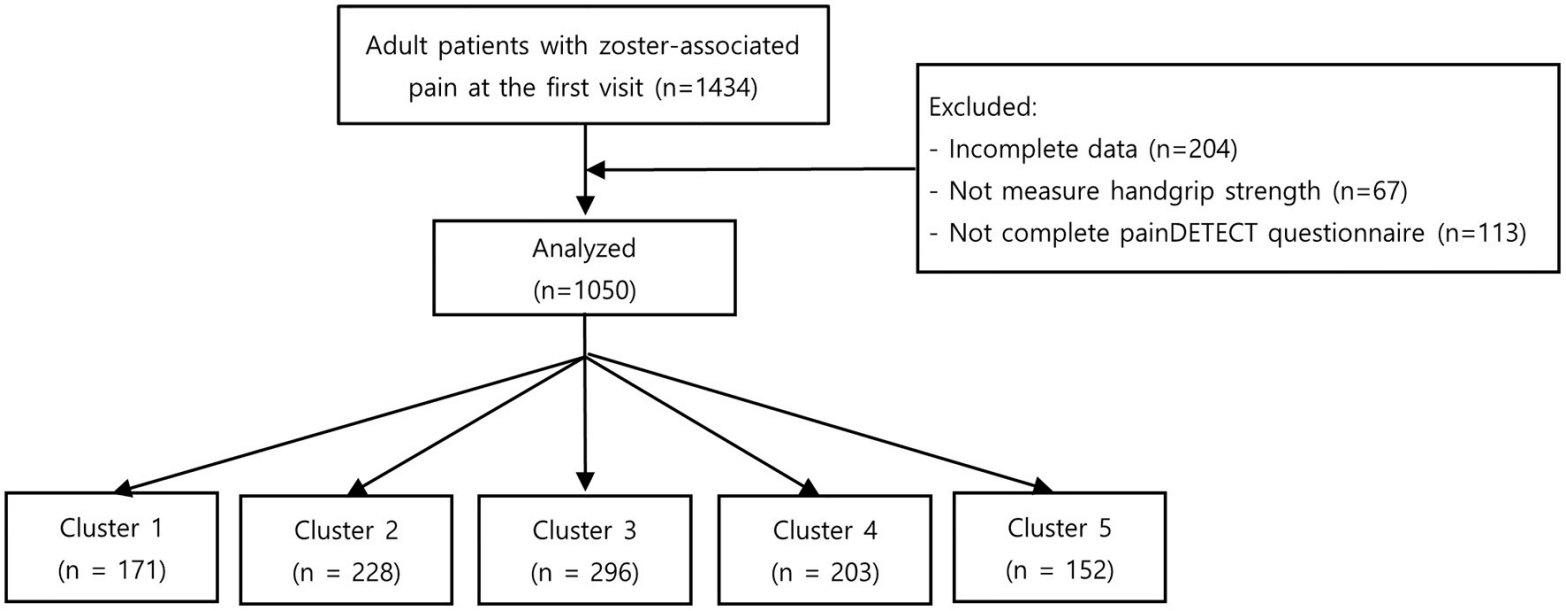
Study flowchart.

During the study period, 1,434 patients visited our clinic for zoster-associated pain. Among these patients, 384 met the exclusion criteria, leaving 1,050 patients in the analysis (Figure 2).

Demographic profiles and pain-related data for these patients are shown in Table 1. Women accounted for more than 50% of the patient group, the mean age was 63.86 years, and the average BMI value was 23.55 kg/m^2^. Cardiovascular disease, diabetes mellitus, and mental health problems were reported for 362, 207, and 166 patients, respectively, and more than 50% of the patients complained of sleep disturbances. The mean average pain score was 5.57 and the maximum pain score was 6.29 on the NRS. A total of 302 patients were assessed at < 1 month after zoster infection, 315 patients had passed 1–3 months, and 433 patients complained of pain for more than 3 months. There were 201 patients with zoster in the trigeminal and facial nerve areas, 167 with zoster in the cervical dermatomes, 546 in the thoracic dermatomes, and 136 in the lumbosacral dermatomes. Approximately 80.6% of patients were taking a gabapentinoid to relieve pain.

**Table 1:** Patient characteristics and pain-related data of 1,050 patients with zoster-associated pain.

**Variables**	**Total (*n =* 1,050)**
**Patient characteristics**	
Age, years	63.86 ± 13.87
Female sex	616 (58.7)
Body mass index, kg/m^2^	23.55 ± 3.13
Cardiovascular disease	362 (34.5)
Diabetes mellitus	207 (19.7)
Mental health problems	166 (15.8)
Sleep disturbance	563 (53.6)
**Pain-related data**	
Pain score, numeric rating scale 0–10	
Average	5.57 ± 2.44
Maximum	6.29 ± 2.41
Pain duration	
< 1 month	302 (28.8)
1–3 months	315 (30.0)
≥ 3 months	433 (41.2)
Dermatomes	
Trigeminal, facial	201 (19.1)
Cervical	167 (15.9)
Thoracic	546 (52.0)
Lumbosacrum	136 (13.0)
Gabapentinoid use	846 (80.6)

Values are presented as mean ± standard deviation (SD) or number of patients (%).

**Figure 3:**
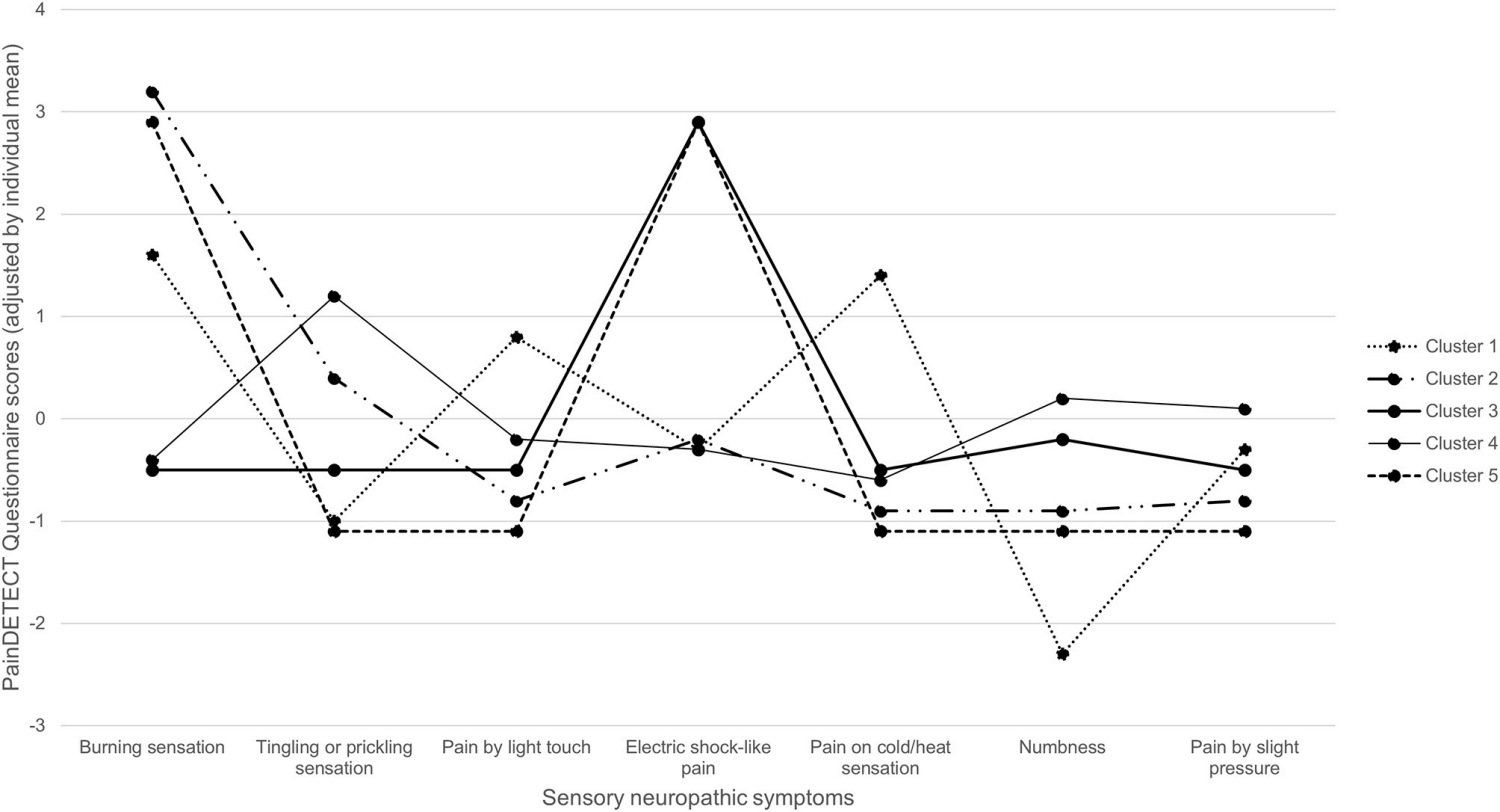
Distribution of sensory symptom profiles of derived five cluster groups.

A cluster analysis was performed using the Korean version of painDETECT to identify the subgroups of patients showing typical sensory neuropathic symptoms as 5 distinct clusters (Figure 3). Patients in cluster 1 complained of all three symptoms: burning sensations, allodynia, and thermal sensitivity, although a lack of numbness was also reported. Patients in clusters 2 and 3 complained of severe burning sensation and electric-shock-like pain, respectively. Cluster 4 patients reported relatively strong tingling pain with an intensity similar to that of most sensory symptoms. Cluster 5 patients reported both burning and shock-like pains.

A comparison was conducted to determine whether there was a difference in the characteristics and pain-related data of the patient groups between the 5 clusters (Table 2). Patient age (cluster 1 vs. 2, *p* = 0.001; cluster 1 vs. 3, *p* = 0.002; cluster 1 vs. 4, *p* = 0.006; cluster 1 vs. 5, *p* = 0.009) and the prevalence of cardiovascular disease (cluster 1 vs. 2, *p* < 0.001; cluster 1 vs. 3, *p* < 0.001; cluster 1 vs. 4, *p* = 0.005; cluster 1 vs. 5, *p* < 0.001) were significantly lower in cluster 1 than those in the other clusters. In addition, clusters 1 and 4 had a relatively high proportion of patients complaining of pain for more than 3 months (chronic) compared to clusters 2 and 3 (cluster 1 vs. 2, *p* = 0.013; cluster 1 vs. 3, *p* = 0.044; cluster 4 vs. 2, *p* = 0.007; cluster 4 vs. 3, *p* = 0.027). However, there were no significant differences in sex, BMI, medical comorbidities including diabetes mellitus and mental health problems, and sleep disturbance between clusters. Also, the average and maximum pain scores on the NRS, the dermatomes to which the lesion belongs, and the use of gabapentinoids were similar between cluster groups.

**Table 2:** Comparison of demographics, pain-related data, and affected dermatomes among the five cluster group.

**Cluster label**	**Cluster 1** **(*n* = 171)**	**Cluster 2** **(*n* = 228)**	**Cluster 3** **(*n* = 296)**	**Cluster 4** **(*n* = 203)**	**Cluster 5** **(*n* = 152)**
**Patient characteristics**					
Age, years*	60.25 ± 14.22^2, 3, 4, 5^	65.37 ± 14.61^1^	64.26 ± 13.12 ^1^	64.25 ± 13.57^1^	64.32 ± 13.66^1^
Female sex	88 (51.5)	132 (57.9)	184 (62.2)	114 (56.2)	98 (64.5)
Body mass index, kg/m^2^	23.81 ± 2.86	23.52 ± 3.07	23.68 ± 3.37	23.69 ± 3.02	23.03 ± 3.01
Cardiovascular disease**	35 (20.5)^2, 3, 4, 5^	89 (39.0)^1^	110 (37.2)^1^	68 (33.5)^1^	60 (39.5)^1^
Diabetes mellitus	29 (17.0)	53 (23.2)	56 (18.9)	38 (18.7)	31 (20.4)
Mental health problems	20 (11.7)	40 (17.5)	56 (18.9)	22 (10.8)	28 (18.4)
Sleep disturbance	95 (55.6)	116 (50.9)	157 (53.0)	107 (52.7)	88 (57.9)
**Pain-related data**					
**Pain score, numeric rating scale, 0–10**					
Average	5.23 ± 2.54	5.46 ± 2.43	5.82 ± 2.39	5.51 ± 2.40	5.73 ± 2.42
Maximum	6.05 ± 2.55	6.35 ± 2.45	6.41 ± 2.32	6.06 ± 2.41	6.57 ± 2.35
**Pain durations**					
< 1 month	49 (28.7)	70 (30.7)	99 (33.4)	45 (22.2)	39 (25.7)
1-3 months	41 (24.0)	78 (34.2)	85 (28.7)	61 (30.0)	50 (32.9)
≥ 3 months**	81 (47.4)^2, 3^	80 (35.1)^1, 4^	112 (37.8)^1, 4^	97 (47.8)^2, 3^	63 (41.4)
**Dermatomes**					
Trigeminal, facial	39 (22.8)	47 (20.6)	55 (18.6)	33 (16.3)	27 (17.8)
Cervical	29 (17.0)	40 (17.5)	35 (11.8)	38 (18.7)	25 (16.4)
Thoracic	83 (48.5)	115 (50.4)	162 (54.7)	108 (53.2)	78 (51.3)
Lumbosacrum	20 (11.7)	26 (11.4)	44 (14.9)	24 (11.8)	22 (14.5)
Gabapentinoid use	141 (82.5)	183 (80.3)	227 (76.7)	164 (80.8)	131 (86.2)

Values are presented as mean ± standard deviation (SD) or number of patients (%).

* Tukey's multiple comparison test (p < 0.05);

** Chi-square *post hoc* difference (p < 0.05). 1, vs. cluster 1; 2, vs. cluster 2; 3, vs. cluster 3; 4, vs. cluster 4; 5, vs. cluster 5.

## Discussion

Patients with zoster-associated pain account for a large proportion of those with neuropathic-like pain symptoms. Despite an increased understanding of the pathophysiological mechanism of neuropathic pain, treatment of zoster-associated pain remains challenging and insufficiently effective. This study was designed to classify patients with zoster-associated pain based on abnormalities in sensory symptoms because it would be ideal and effective to stratify patients according to the pain mechanism (15).

Distinct sensory abnormalities associated with pain perceived by patients can be self-assessed, and they are largely divided into spontaneous sensory sensation (burning, tingling, and electric-shock-like pain), stimulus-evoked positive sensory symptoms (pain evoked by light touch, thermally evoked pain, and pressure-evoked pain), and stimulus-evoked negative sensory symptom (numbness) (7, 16). The variations in the mechanisms of symptom generation are explained by plastic changes in the central nervous system and remaining peripheral nociceptors (6, 17).

An analysis of patients with zoster-associated pain on the basis of sensory phenotype found that patients in cluster 1 suffered primarily from thermally evoked pain and mechanical allodynia, but not numbness. Allodynia is due to central spinal cord sensitization and is induced by activation of touch-sensitive cutaneous Aβ-fibers that terminate in synapses of nociceptive second-order neurons in the central nervous system (18). Thermally evoked pain is due to peripheral sensitization of nociceptive afferents; it is characteristic of hyperactive and sensitized cutaneous nociceptors (19). However, numbness is a sensory symptom that involves a loss of afferent function (11). Cluster 1 patients therefore have irritable nociceptors and their skin innervation is intact (20). In studies of patients with peripheral neuropathic pain, it was possible to predict that thermally evoked pain and allodynia would have an analgesic effect by applying 8% topical capsaicin patches (21) and intracutaneous botulinum toxin (22). Patients in cluster 2, 3, and 5 suffered spontaneous pain without cutaneous hypersensitivity and marked sensory deafferentation, caused by ectopic neuronal firing within the nociceptive pathways and secondary sensitization of central nociceptive neurons (11). Abnormal expression of voltage-gated sodium channels is associated with ectopic nerve activity (23). An animal study has shown increases and changes in voltage-gated ion channels at impaired peripheral nerves in rats with varicellar zoster virus. These sensitized pain behaviors were reversed by sodium channel blockers (24). Peripheral analgesic lidocaine patches selectively block sodium channels of small damaged peripheral nerve fibers without severe systemic adverse effects. Therefore, in this case, applying topical lidocaine is appropriate for the control of localized peripheral neuropathic pain (25). Patients in cluster 4 tended to experience relatively strong tingling sensations, but were also characterized by relatively mild abnormalities due to the overlap of pathophysiological mechanisms. There are two possible causes of these symptom constellations. First, these patients perceived all neuropathic pain at similar frequencies and intensities. The second possibility is that these patients are people who tend to answer in a similar manner. These patients responded similarly to all questions because they could not discriminate between the abnormalities in sensory symptoms. Such patients should be excluded from the clinical trial of a medication that targets specific pathophysiologies.

In the present study, the patients belonging to cluster 1 were younger than those of the other clusters. Although results conflicted depending on the animal model and experimental stimuli, preclinical data suggest that advanced age may be related to increased pain sensitivity (26). However, the relationship between age and pain sensitivity is unclear in zoster-related pain. Moreover, patients in cluster 1 reported experiencing pain for a longer time, although they were younger. Age may therefore not be an independent factor associated with specific sensory symptoms. In the current study, some differences in sensory symptoms according to duration of zoster-associated pain were also observed. In chronic pain conditions, the alteration of central processing of pain is a major contributing factor in pain symptoms (6). While previous studies (11, 12) were conducted primarily on PHN patients, our study included all periods of pain, with more than half of patients reporting acute and subacute periods. Tissue inflammation and destruction, activation of nociceptive neurons, abnormal impulses, and neural injury during the early infection period cause a strong nociceptive barrage, creating acute pain and allodynia (27). Considering the complexity of zoster-associated pain, this study confirms different therapeutic approaches are needed to manage pain according to their pain duration in this population.

This study has several limitations. As a retrospective study conducted in a single center, selective bias or information bias is possible. PainDETECT is a reliable questionnaire to assess differences in sensory perception intensity, but there is a paucity of interpretation of evoked pain and negative symptoms. Also, pain perceptions are subjective and could be confounded by personal factors (7). To minimize these shortcomings, the scores of the questionnaire were adjusted by individual means. A longitudinal study would be necessary to proceed with clustering in an objective psychophysical approach (e.g., Quantitative sensory testing (QST) or morphological data from skin biopsies). In addition, we used an actual clinical practice model in which the majority of attending patients had undergone some interventional treatments or had been prescribed medications, including gabapentinoids. This class of medications modulates pain processing and central sensitization. However, because there was no difference in the ratio of gabapentinoid use for each subgroup, this factor would not have had a significant impact on the interpretation of the results.

## Conclusion

In conclusion, despite the common cause of the pain, some differences in the distribution of sensory profiles were evident among patients with zoster-associated pain. In particular, a subgroup with younger patients who experienced longer durations of pain reported specific and distinct positive symptoms, such as burning sensations and allodynia. Unlike patients with acute or subacute pain, patients with chronic pain were associated with diverse sensory symptom profiles. These results indicate that patients can be subgrouped according to patient-reported sensory symptoms of zoster-associated pain, although the central and peripheral mechanisms of pain vary among subjects. This approach may help determine the most appropriate pain treatment for each individual of this population.

## Data availability statement

The raw data supporting the conclusions of this article will be made available by the authors, without undue reservation.

## Ethics statement

The studies involving human participants were reviewed and approved by the Institutional Review Board of Yonsei University Health System, Seoul, Republic of Korea. Written informed consent for participation was not required for this study in accordance with the national legislation and the institutional requirements.

## Author contributions

Conceptualization, writing—review and editing, and supervision: SHK and KBY. Software and validation: HJK and MK. Formal analysis and data curation: HJK and YSY. Writing—original draft preparation: HJK and SHK. Project administration and methodology: HJK. Funding acquisition: SHK. All authors contributed to the article and approved the submitted version.
